# Hypertension and diabetes in Zanzibar – prevalence and access to care

**DOI:** 10.1186/s12889-020-09432-8

**Published:** 2020-09-04

**Authors:** Jutta M. Adelin Jorgensen, Kaya Helene Hedt, Omar Mwalim Omar, Justine I. Davies

**Affiliations:** 1 Mnazi Mmoja Referral Hospital, Kaunda Rd, Vuga, Po Box 3793, Zanzibar, Tanzania; 2 Department of Public Health, University of Copenhagen, Copenhagen, Denmark; 3 University of North Carolina at Chapel Hill, Chapel Hill, USA; 4 Head of NCD unit, Zanzibar Ministry of Health, Zanzibar, Tanzania; 5 Institute of Applied Health Research, University of Birmingham, Birmingham, UK; 6 MRC/Wits Rural Public Health and Health Transitions Research Unit, School of Public Health, University of Witwatersrand, Johannesburg, South Africa; 7 King’s Centre for Global Health, King’s College, London, UK

**Keywords:** Hypertension, Diabetes, Mental illness, Access to care, Zanzibar, Tanzania, Sub-Saharan Africa

## Abstract

**Background:**

Cardiovascular diseases are among the most common causes of hospital admissions and deaths in Zanzibar. This study assessed prevalence of, and antecedent factors and care access for the two common cardiovascular risk factors, hypertension and diabetes, to support health system improvements.

**Methods:**

Data was from a population based nationally representative survey. Prevalence of hypertension was defined as systolic blood pressure ≥ 140 mmHg, diastolic blood pressure ≥ 90 mmHg or a self-reported diagnosis of hypertension; diabetes was defined as a fasting blood glucose ≥6.1 mmol/L or a self-reported diagnosis of diabetes. Care-cascades for hypertension and diabetes were created with four stages: being tested, diagnosed, treated, and achieving control.

Multivariable logistic regression models were constructed to evaluate individual-level factors – including symptoms of mental illness - associated with having hypertension or diabetes, and with progressing through the hypertension care cascade. Whether people at overt increased risk of hypertension or diabetes (defined as > 50 years old, BMI > 30 kg/m2, or currently smoking) were more likely to be tested was assessed using chi squared.

**Results:**

Prevalence of hypertension was 33.5% (CI 30.6–36.5). Older age (OR 7.7, CI 4.93–12.02), some education (OR 0.6, CI 0.44–0.89), obesity (OR 3.1, CI 2.12–4.44), and raised fasting blood glucose (OR 2.4, CI 2.38) were significantly independently associated with hypertension.

Only 10.9% (CI 8.6–13.8) of the entire hypertensive population achieved blood pressure control, associated factors were being female (OR 4.8, CI 2.33–9.88), formally employed (OR 3.0, CI 1.26–7.17), and overweight (OR 2.5, CI 1.29–4.76).

The prevalence of diabetes was 4.4% (CI 3.4–5.5), and associated with old age (OR 14.1, CI 6.05–32.65) and almost significantly with obesity (OR 2.1, CI 1.00–4.37). Only 11.9% (CI 6.6–20.6) of the diabetic population had achieved control.

Individuals at overt increased risk were more likely to have been tested for hypertension (chi2 19.4) or diabetes (chi2 33.2) compared to the rest of the population.

Symptoms of mental illness were not associated with prevalence of disease or progress through the cascade.

**Conclusion:**

High prevalence of hypertension and suboptimal management along the care cascades indicates a large unmet need for hypertension and diabetes care in Zanzibar.

## Background

Cardiovascular diseases (CVD) are traditionally considered diseases common to high income countries (HIC), but nonetheless are an increasing health concern in low- and middle-income countries (LMIC).

High blood pressure is quantitatively the most important risk factor for CVD, and Sub Saharan Africa (sSA) has the highest prevalence of hypertension in the world [1]. Country-specific data from the United Republic of Tanzania (to which Zanzibar belongs) shows cardiovascular diseases are increasingly contributing to the number and quality of life lost,^1^ but there is no separate published data from Zanzibar despite its status as a semi-autonomous part of the republic where provision of health services falls under the Government of Zanzibar. Additionally, the epidemiological and demographic landscape differs between mainland Tanzania and Zanzibar with the latter, for example, having low prevalence and incidence of HIV and TB as well as malaria. Other differences include a much higher urbanization rate in Zanzibar with just below half of the population of 1.3 million in urban areas, and a lower GDP per capita at 907 USD (non-adjusted), with 43.5% of the population living below the international poverty line of 1.9 USD per day [2] and a concentration of the poor in rural areas.

The epidemiological pattern of hypertension and diabetes may differ according to the stage of health transition that a country is going through, thus it is important to identify local drivers of risk factors as well as drivers of disease control in Zanzibar independent from Tanzania. In order for the government of Zanzibar to plan for management and prevention of hypertension and diabetes, and put in place health system (public health and health service) measures to prevent conditions and adequately treat those people who already have them, data on disease prevalence and risk factors, and health service performance is essential.

A cascade of care approach can be utilized to identify where gaps in care provision (from testing to diagnosis, treatment, and control) occur. Pooled analysis of data from individual surveys shows very low performance of health systems in sSA with gaps in care provision throughout the care cascade leaving > 90% of people with hypertension, and close to 80% of people with diabetes, without adequate control [3–5].

When considering testing, in an under-resourced setting, disease detection may initially be best deployed to detect people who are at high risk of conditions [6]. It has been shown that several factors, including increased age, high BMI, and smoking, are associated with increased risk of hypertension and diabetes. These are all individual level factors that a health care provider can easily identify, and which could be used to trigger testing. However, the extent to which these factors are of use to guide testing in practice needs further exploration.

Poor mental health is increasingly being recognized as an issue in LMIC settings, and research predominantly done in HIC suggests it affects prevalence of cardiovascular disease risk factors as well as access to treatment [7, 8]. In HICs, presence of poor mental health results in worse disease control [9] for these people, who are likely to require increased effort to receive adequate care. However, the relationships between markers of poor mental health and prevalence of and access to treatment for cardiovascular disease risk factors are not well explored in low- and middle-income country settings.

Although previous studies have looked at prevalence of cardiovascular disease risk factors in Sub Saharan Africa and used a cascade of care approach to assess health service provision, this has not been done previously in Zanzibar. Additionally, we are not aware of other studies that have looked at the relationship between mental health conditions and prevalence of and care for hypertension or diabetes in a sSA setting.

The objectives of this analysis are threefold: to explore the epidemiology of hypertension and diabetes in Zanzibar, to assess the health services’ effectiveness to detect and successfully treat these conditions – in particular to examine whether or not people at increased risk of conditions had been tested for them - and to identify individual level characteristics, including prevalence of poor mental health, associated with developing these conditions and being tested/diagnosed/treated/controlled.

## Methods

### Data

Data from the National Non-Communicable Disease Risk Factor Survey, a population based, nationally representative, cross-sectional survey done in 2011 was used. The survey was the first of its kind in Zanzibar. The survey design and methods are described in detail elsewhere [10]. In brief, a modified version of the World Health Organization instrument for stepwise approach to noncommunicable disease risk factors surveillance (STEPS) was applied. Multi-staged cluster sampling methods were used to recruit a random and representative sample of the general population aged 25–64 years, using households as the final sampling frame and randomly selecting individual participants at household level. Individual participant data including socio-demographic characteristics, behavioral risk factors (physical activity, smoking, alcohol use, and diet), previous history of hypertension and diabetes, history of taking medication for diabetes, hypertension, or raised cholesterol, symptoms of mental illness; anthropometric measurements of weight and height; blood pressure measurement; and capillary blood for biochemical analysis using point of care devices (fasting blood sugar and total cholesterol) were collected through standardized procedures and with the participant being instructed to fast.

The 12 item General Health Questionnaire GHQ-12 which has previously been used in sSA [11] was used to identify symptoms of minor psychiatric disorders after having been translated into Swahili and back-translated to resolve discrepancies in conceptual equivalence.

BP was measured (Omron M2) three times during a single visit to the participant’s home, after 30 min of rest and with at least 3 min between each measurement; the average of second and third reading was used for analysis. A minority of participants did not have three consecutive BP measurements done; in those the average of available readings was used. Height and weight were measured with participants wearing light clothing and being bare foot, using generic scales and measuring tape applied to a rod, purchased locally. Blood glucose and total cholesterol (AccuTrend Plus GCT meter, Roche Diagnostics) were measured in the morning hours the day after the survey interview had taken place, and only on (self-reported) fasting participants.

### Outcome variables

#### Hypertension

Prevalent hypertension (HTN) was defined as having a systolic blood pressure (BP) ≥140 mmHg or diastolic BP ≥90 mmHg measured during the survey, or a self-reported previous diagnosis of hypertension. Previous testing for HTN was defined as a positive response to being asked if participants had ever had a BP measurement done. Amongst those who recalled having been tested for hypertension, those who reported having been diagnosed by a doctor or health care worker were defined as previously diagnosed, amongst those previously diagnosed, those who reported receiving prescribed medication in the past 2 weeks and/or advice on behavior changes, were defined as treated. Among those treated, controlled hypertension was defined as having a SBP < 140 mmHg and DBP < 90 mmHg measured during the survey.

### Diabetes and impaired fasting glucose

The IDF diagnostic values were used. Impaired fasting glucose (IFG) was defined as a fasting blood glucose (FBG), measured on capillary whole blood, ≥ 5.6 mmol/L and < 6.1 mmol/L. Prevalent diabetes mellitus (DM) was defined as having a FBG ≥ 6.1 mmol/L or a self-reported diagnosis of diabetes. Raised fasting blood glucose was defined as FBG ≥ 5.6 mmol/L. Previous testing for diabetes was defined as a positive response to being asked if the participant had ever had a blood glucose measurement done. Amongst those who recalled having been tested for diabetes, those who reported having been diagnosed by a doctor or health care worker were defined as diagnosed, and among those diagnosed, those who reported receiving insulin, prescribed oral medication in the past 2 weeks and/or advice for diabetes were defined as treated. Amongst those who were treated, controlled diabetes was defined as having a FBG < 5.6 mmol/L measured during the survey.

IFG and diabetes were merged to construct two categories (1) normal fasting blood glucose (2) raised fasting blood glucose, for the analysis of variables associated with hypertension.

### Care cascades for hypertension and for diabetes

A care cascade approach was used to describe access to care for hypertension and diabetes [12]. The method for constructing the care cascade was the same for hypertension and diabetes using individual participant data. Cascades were constructed using two methods. First, the percentage of the total population with prevalent disease who achieved each step of the cascade was presented. Second, the losses at each stage of the cascade using the number of people who had reached the previous step as the denominator was presented. Entry into each step of the cascade was dependent on having reached the stage before it. An illustration of the cascade is enclosed in the additional material (Additional file 1).

### Independent variables

#### Socio-demographic (risk) factors

Age was categorized as 25–34 years, 35–49 years, and 50–64 years. The highest level of education achieved was defined as (1) no formal education (2) some schooling but less than secondary (10 years) completed (3) secondary school or above completed. Data on occupation was collected in pre-coded categories (STEPS standard version) and re-categorized into (1) no formal work (included housewives) (2) self-employed (3) formally employed (wage work) whether by government or non-government employer. Residency was categorized as urban or rural according to the Office of the Chief Government Statistician in Zanzibar.

#### Behavioral risk factors and BMI

Physical activity was defined as low, moderate, or high depending on the sum of intensities of physical activity during work, recreation, and transportation, calculated using the unit metabolic equivalent minutes (METs). Physical inactivity was defined as not meeting the minimum standard of WHO of 600 METs per week. Sedentary time during waking hours was based on self-reported values and, informed by relevant literature on the association between outcomes and sedentary time, results were dichotomized into a binary variable with a threshold of 3 h per day [13].

Overweight was defined as a body mass index (BMI) of ≥25.0 and ≤ 30 kg/m2, and obesity as BMI > 30.0 kg/m2. Smoking status was categorized as current smokers, former smokers, and those who never smoked. Alcohol use was excluded from the analysis due to the low number of drinkers in the surveyed population, and diet was excluded as the vast majority of the population consumed fruits and/or vegetables at very low quantity and frequency, while salt and sugar intake was not assessed.

#### Mental illness

Minor (non-psychotic) psychiatric morbidity was assessed using the 12- item General Health Questionnaire (GHQ), which is a widely used screening tool for minor psychiatric illnesses [14]. Respondents initially responded to each item using a four-point Likert scale indicating presence or absence of symptom. During analysis, responses were collapsed to into a two-point scale (GHQ method), scoring 0 or 1 on each item. The sum of all items gave a total score out of 12; a conservative cut-off was set at a sum of 4 or more to signify presence of psychiatric morbidity mainly in the axis of depression/anxiety.

#### Raised cholesterol

Raised blood cholesterol was defined as either being on medication for raised cholesterol or having a measurement of total cholesterol ≥5 mmol/L. HDL-LDL ratio was not assessed.

#### Increased risk for diabetes or hypertension

‘At increased risk’ for diabetes or hypertension was defined based on whether an individual had overt characteristics that might signify increased risk and that a healthcare professional in an under-resourced setting could detect without access to patient records or diagnostic tests. The definition included any one of the following: being above 50 years, BMI at or above 25 kg/m2, or currently smoking.

### Ethics

The National NCD risk factor survey received ethical approval from Zanzibar Medical Research Ethical Committee (ZAMREC), Ministry of Health, and by the Office of 2nd Vice President, Revolutionary Government of Zanzibar. All participants had given their informed consent to participate in the survey. Approval for publication of this paper was obtained from Zanzibar Ministry of Health following ordinary procedures.

### Analysis

Analyses are restricted to participants with non-missing, measured, variables. Among these, due to the skip-to-next-pattern of the electronic data collection tool used, responses which were negative (for instance ‘have you ever been told by a health worker that you have hypertension’), led automatically to skip of following related questions (such as ‘are you currently on treatment for hypertension’), and hence left as a missing entries. Therefore, for selfreported variables apart from age, missing entries were assumed to be negative responses. In the analyses to estimate the losses at each stage of the care cascade, these missing/negative values were omitted from the analyses as described above.

Using the 2012 census data, standardization based on age and sex was done to the distribution of the Zanzibar adult population, and sample weights were incorporated to adjust for unequal probability of selection at population level.

For the descriptive analysis, unweighted numbers but weighted proportions are displayed. Categorical variables are expressed as percentages, and continuous variables by mean and standard error (SEM).

(Univariable logistic regression analyses are enclosed in additional material (see Additional file 4, 5 and 6).)

Multivariable logistic regression with forced entry using weighted variables was used to test associations between prevalence of diabetes or hypertension and independent variables age; sex; area of residency; education; employment; tobacco use; BMI; sedentary time; symptoms of mental ill health; and high cholesterol. For hypertension, raised fasting blood glucose (measured) was furthermore included, given the effects of uncontrolled/poorly controlled blood sugar on hypertension [15].

Success in achieving each step of the cascade was regressed against the above variables, for the hypertension cascade, using a known diagnosis of diabetes instead of measured raised fasting blood glucose, because an existing diagnosis could lead to more attention from health care providers and affect progress through the cascade. Study-measured high cholesterol was not included as a co-variable, given this is unlikely to affect transit through the cascade.

Finally, univariable analyses comparing the increased-risk group with the low-risk group was done for the three outcomes (1) prevalence of disease (2) having been tested, and (3) achieving adequate control, using Pearson chi square.

Analyses were performed on weighted data using Stata v.14.1 (Stata) with *p* value < 0.05 considered to represent statistical significance, and confidence interval at 95% for all statistical analyses.

## Results

**Fig. 1:**
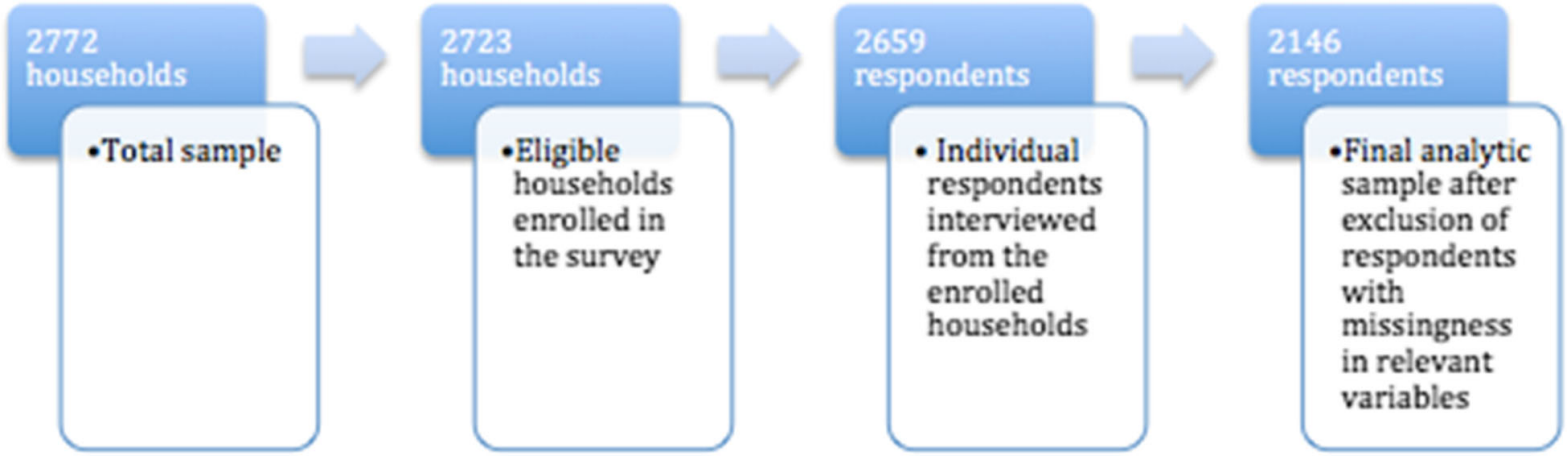
Analytic sample

2772 households were initially sampled for the survey. Out of these, 2659 (97.6%) households had one member randomly selected to participate in the study. After excluding participants with missing information from the dataset, 2146 (80.7%) respondents remained (Fig. 1).

Participant characteristics are shown in Table 1.

**Table 1:** Respondent characteristics

Characteristics [mean (SEM) or unweighted n (%)]	Total	Women	Men
	2146 (100%)	1354 (53.1%)	792 (46.9%)
Age (years) [mean]	38.4 [0.30]	37.6 [0.34]	39.2 [0.51]
BMI (kg/m2) [mean]	24.5 [0.18]	25.3 [0.24]	23.5 [0.25]
Blood pressure (mmHg), measured			
Systolic blood pressure [mean]	130.2 [0.72]	127.7 [1.00]	132.9 [1.03]
Diastolic blood pressure [mean]	77.8 [0.44]	77.7 [0.59]	79 [13.9]
BP ≥140/90 mmHg	340 (29.8%)	221 (28.5%)	119 (31.4%)
Age groups			
25–34 years	717 (40.0%)	469 (41.1%)	248 (38.7%)
35–49 years	897 (43.0%)	593 (45.1%)	304 (40.7%)
50–64 years	532 (17.0%)	292 (13.7%)	240 (20.6%)
Blood glucose (mmol/L), measured			
Fasting Blood Glucose (FBG) [mean]	4.41 [0.05]	4.36 [0.08]	4.48 [0.07]
Impaired FBG	65 (2.5%)	41 (1.9%)	24 (3.2%)
Diabetic FBG (at or above 6.1 mmol/L)	99 (3.9%)	61 (3.7%)	38 (4.0%)
Marital status			
single, never married	138 (9.3%)	60 (0.06%)	78 (13.3%)
married or cohabiting	1741 (81.3%)	1060 (80.2%)	681 (82.6%)
divorced or widowed	267 (9.4%)	234 (14.2%)	33 (0.04%)
Residence			
Rural	1337 (50.2%)	826 (49.4%)	511 (51.1%)
Urban	809 (49.8%)	528 (50.6%)	281 (48.9%)
Education			
No formal education	554 (18.9%)	418 (24.2%)	136 (13.0%)
Some primary or secondary education	958 (48.8%)	574 (46.9%)	384 (50.9%)
Secondary education or above completed	634 (32.3%)	362 (28.9%)	272 (36.0%)
Employment status			
No formal employment	846 (34.6%)	769 (56.9%)	77 (9.5%)
Self-employed	972 (45.5%)	430 (29.4%)	542 (63.8%)
Formally employed	328 (19.8%)	155 (13.8%)	173 (26.7%)
Tobacco use			
Never smoked	1850 (85.9%)	1310 (97.8%)	540 (72.4%)
Current smoker	139 (7.3%)	31 (1.4%)	126 (14.0%)
Former smoker	157 (6.8%)	13 (0.8%)	126 (13.6%)
Sedentary time/day			
Less than 3 h	1459 (69.1%)	916 (68.3%)	543 (70.1%)
3 h or more	687 (30.9%)	438 (31.7%)	249 (29.9%)
Physical activity level/week			
At or above 600 METs	1488 (69.4%)	856 (63.3%)	632 (79.8%)
Below 600 METs	656 (30.6%)	496 (36.7%)	160 (20.2%)
BMI classification			
Underweight	153 (8.1%)	110 (8.5%)	70 (7.9%)
Normal weight	1180 (54.7%)	659 (48.8%)	494 (61.2%)
Overweight	474 (22.3%)	306 (22.2%)	168 (22.3%)
Obese	339 (14.9%)	279 (20.4%)	60 (8.7%)
Total cholesterol			
Raised (≥5 mmol/L)	593 (25.8%)	446 (31.0%)	147 (19.8%)
Previous diagnosed with diabetes	40 (1.7%)	20 (1.2%)	20 (2.2%)
Mental ill health	152 (6.9%)	118 (8.7%)	34 (4.9%)
Has hypertension (as per study definition)	813 (33.5%)	515 (35.5%)	298 (32.8%)
Has diabetes (as per study definition)	114 (4.7%)	69 (4.6%)	45 (4.7%)
Previously had BP measured	1151 (50.0%)	872 (69.9%)	279 (32.3%)
Previously had FBG measured	382 (16.4%)	249 (17.9%)	133 (14.5%)

Data are displayed as mean (and standard error) or percentage (and number of participants). All percentages are calculated using weighted variables. Numbers of participants are non weighted. Study definition of hypertension: Previously diagnosed with hypertension by health care worker, or a measured SBP ≥ 140 mmHg or DBP ≥ 90 mmHg during the survey. Study definition of diabetes: Previously diagnosed with diabetes by health care worker, or a measured FBG ≥ 6.1 mmol/L during the survey

### Hypertension

The crude prevalence of hypertension in the population was 33.5% (CI 30.58–36.52). Factors significantly associated with hypertension on multivariable analysis were increasing age (OR 2.1, CI 1.43–3.08 for the 35–49 year group, and OR 7.7, CI 4.93–12.02 for the 50–64 years age group), obesity (OR 3.1, CI 0.40–1.17), and raised fasting blood glucose (OR 2.4, CI 1.34–4.22). Prevalence of hypertension was less common in those with some as compared with no formal education (OR 0.6, CI 0.44–0.89 for some primary/secondary education) (Table 2).

**Table 2:** Multivariable regression analysis

	Hypertension				Diabetes	
	OR	95% CI	*p*-value	OR	95% CI	*p*-value
**Sex**						
Male	1.0			1.0		
Female	0.9	(0.63, 1.29)	0.570	1.0	(0.55, 1.85)	0.985
**Age**						
24–34 years	1.0			1.0		
35–49 years	2.1	(1.43, 3.08)	< 0.001	3.2	(1.33, 7.69)	0.009
50–64 years	7.7	(4.93, 12.02)	< 0.001	14.0	(6.05, 32.65)	< 0.001
**Residence**						
Rural	1.0			1.0		
Urban	0.8	(0.57, 1.10)	0.167	1.4	(0.80, 2.41)	0.247
**Education**						
No formal education	1.0			1.0		
Some primary/secondary school	0.6	(0.44, 0.89)	0.008	1.6	(0.82, 2.66)	0.174
Secondary school or above complete	0.8	(0.54, 1.22)	0.306	2.0	(0.98, 4.19)	0.058
**Employment**						
No formal or self employment	1.0			1.0		
Self employed	0.9	(0.63, 1.22)	0.422	1.7	(0.85, 3.26)	0.139
Formally employed	0.8	(0.52, 1.31)	0.414	1.3	(0.58, 2.76)	0.563
**Tobacco use**						
Never smoked	1.0			1.0		
Former smoker	1.5	(0.73, 2.87)	0.288	0.8	(0.32, 2.14)	0.692
Current smoker	0.7	(0.40, 1.17)	0.168	0.9	(0.37, 2.34)	0.881
**BMI**						
Normal or underweight	1.0			1.0		
Overweight	1.3	(0.94, 1.91)	0.111	1.9	(0.99, 3.49)	0.056
Obese	3.1	(2.12–4.44)	< 0.001	2.1	(1.00, 4.37)	0.051
**Fasting blood glucose**						
Normal fasting blood glucose	1.0					
Raised fasting blood glucose	2.4	(1.34, 4.22)	0.003	–	–	–
**Total cholesterol**						
Normal cholesterol	1.0					
High cholesterol	1.4	(0.80, 2.35)	0.251	2.0	(0.89, 4.43)	0.091
**Sedentary time**						
Sedentary for less than 3 h/day	1.0					
Sedentary for at least 3 h/day	1.0	(0.76, 1.39)	0.854	0.6	(0.36, 1.09)	0.099
**Mental illness**						
No signs of mental illness	1.0					
Mental illness present	1.6	(0.83, 3.18)	0.155	1.7	(0.64, 4.46)	0.290

Estimated Odds Ratios for relationship between independent variables and having hypertension or having diabetes, among total surveyed population, weighted using survey population weights (unweighted n = 2146)

**Fig. 2:**
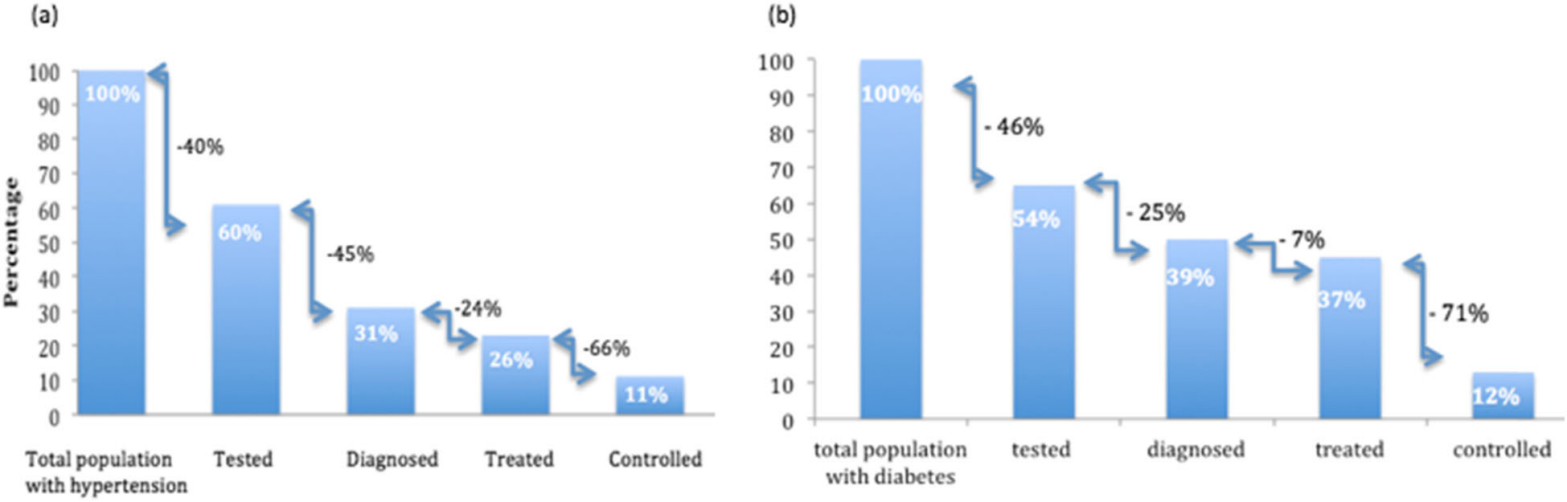
Hypertension and diabetes care cascades. Progress through the (**a**) hypertension and (**b**) diabetes care cascade. Arrows indicate the losses (the relative proportion that does not reach the next stage of the cascade; denominator depends on having reached previous stage), while bars indicate the absolute proportion of all people with disease that progress through the cascade

Among those defined as having hypertension, 59.6% (CI 56.0–65.8) reported having previously had their pressure measured; 51.1% (CI 44.4–57.8) of these had received a diagnosis of hypertension; 79.8% (CI 73.5–85.0) of those diagnosed were currently receiving treatment; and 28.3% (CI 21.4–36.3, *n* = 110) of those treated had achieved BP control. Figure 2(a) summarizes the gaps between the overall HTN population and testing, testing and diagnosing, diagnosing and treating, and treating and receiving control.

The largest relative gap along the hypertension control cascade is between being treated and achieving control. The largest absolute gaps in terms of numbers lost appear at the first two stages with being tested and being diagnosed.

Table 3 shows characteristics associated with progress through the hypertension cascade. Older age was associated with better achievements of most stages of the cascade (from testing to treatment), while younger age was the only variable associated with higher OR of moving from treatment into control (OR 0.1 for eldest age group as compared to youngest, CI 0.03–0.34). Being female (OR 5.3, CI 2.87–9.62), older age (OR 2.1, CI 1.05–4.01 for oldest age group), being educated (OR 3.4, CI 1.67–7.04 for highest education), being formally employed (OR 3.3, CI 1.36–8.12), and being obese (OR 2.4, CI 1.00–3.68), were all associated with increased odds ratio for having been tested for hypertension.

**Table 3:**
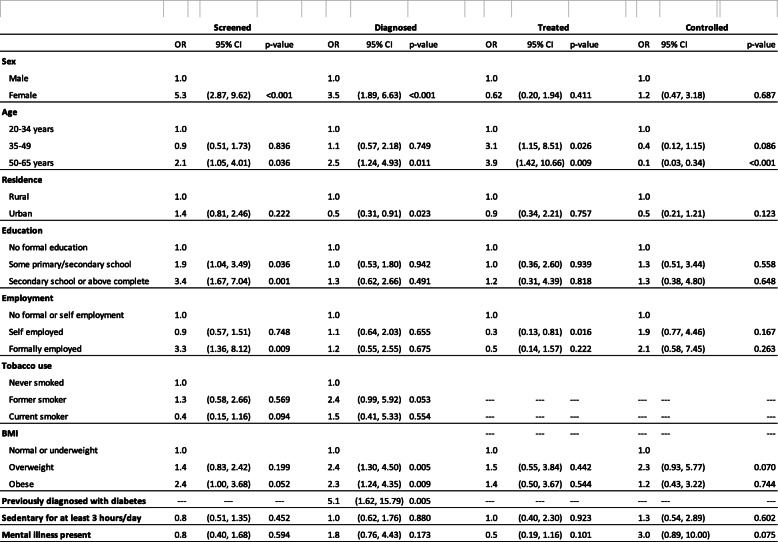
Progress through the hypertension care cascade

Estimated Odds Ratios for relationship between individual level variable, and progressing to next stage in the care cascade. Each variable adjusted for all other variables

Moving from having been tested to being diagnosed was associated with being female (OR 3.54, CI 1.89–6.63), older age (OR 2.47, 1.24–4.93), overweight or obese (OR 2.32, CI 1.24–4.35 for obesity), former smoker (OR 2.42, CI 0.99–5.92), and having a known diagnosis of diabetes (OR 5.05, CI 1.62–15.79), and negatively associated with living in urban area (OR 0.53, CI 0.31–0.91), all *p* < 0.05.

Moving from diagnosis into treatment was associated with older age (OR 3.88, CI 1.42–10.66) and negatively associated with self-employment (OR 0.32, CI 0.13–0.81).

The total gap in hypertension control care (the sum of hypertensive individuals who did not achieve blood pressure control [defined as SBP below 140 mmHg and DBP below 90 mmHg] no matter where along the care cascade they were lost) was 89.1%, i.e.: 10.9% of all people with hypertension were controlled to target. Control among all individuals with hypertension was associated with being woman (OR 4.8 CI 2.33–9.88), formally employed (OR 3.0 CI 1.26–7.17), and overweight (OR 2.5 CI 1.29–4.76) (Table 4).

**Table 4:** Control of blood pressure among all individuals with hypertension

	Control among all HTN		
	OR	95% CI	p-value
**Sex**			
Male	1.0		
Female	4.8	(2.33–9.88)	< 0.001
**Age**			
20–34 years	1.0		
35–49	0.6	(0.30–1.12)	0.106
50–65 years	0.6	(0.28–1.29)	0.187
**Residence**			
Rural	1.0		
Urban	0.6	(0.28–1.07)	0.077
**Education**			
No formal education	1.0		
Some primary/secondary school	1.1	(0.50–3.88)	0.391
Secondary school or above complete	1.5	(0.59–3.88)	0.391
**Employment**			
No formal or self employment	1.0		
Self employed	1.9	(0.92–3.87)	0.081
Formally employed	3.0	(1.26–7.17)	0.013
**Tobacco use**			
Never smoked	1.0		
Former smoker	0.6	(0.19–1.89)	0.388
Current smoker	0.4	(0.09–1.93)	0.264
**BMI**			
Normal or underweight	1.0		
Overweight	2.5	(1.29–4.76)	0.007
Obese	1.9	(0.86–4.11)	0.115
**Previously diagnosed with diabetes**	0.6	(0.21–1.65)	0.312
**Sedentary for at least 3 h/day**	0.8	(0.47–1.45)	0.497
**Mental illness present**	2.0	(0.81–4.70)	0.135

Estimated Odds Ratios for relationship between individual level variable, and achieving adequate BP control, among all people with hypertension (n = 813). Each variable adjusted for all other variables

56.4% (1211 of 2146) of the population were identified as being overtly at increased risk of diabetes or hypertension due to age > 50 years, BMI > 25 kg/m2, or currently smoking. In these participants, prevalence of HTN was twice as high as in the rest of the population (44.8% versus 22.6%, Pearson chi2 116.6, *p* < 0.001). A significantly higher percentage among those with increased risk had been tested for hypertension than among those at lower risk (54.8% versus 44.8%, Pearson chi2 21.4 *P* < 0.001), while there was no significant difference among the two groups in terms of achieving adequate BP control (Table 5).

**Table 5:** Progress through the care cascades for people with overt increased risk as compared with the rest

	At increased risk	No increased risk	chi2	p
**Total population**	1211 (56.4%)	935 (43.6%)	–	–
Had been tested for HTN	700 (54.8%)	451 (44.8%)	21.4	< 0.001
Had been tested for DM	276 (22.8%)	106 (11.3%)	47.3	< 0.001
Had been tested for both HTN and DM	254 (21.0%)	96 (10.3%)	44.3	< 0.001
Had achieved control of BP	56 (31.5%)	16 (36.4%)	0.4	0,534
Had achieved control of BG	12 (37.5%)	1 (20.0%)	–	–
Had HTN	592 (44.8%)	221 (22.6%)	116.6	< 0.001
Had DM	94 (7.2%)	20 (2.0%)	32.3	< 0.001

Proportion of people with increased risk that had HTN or DM, had been tested for HTN, DM, or both HTN and DM, and, among the subgroup which had HTN or DM, had achieved control of blood pressure (BP) or blood glucose (BG)

### Diabetes

The crude prevalence of diabetes (defined as either previously diagnosed, or FBG at the time of the survey at or above 6.1 mmol/L) was 4.4% (CI 3.4–5.5). Diabetes was significantly associated with increasing age (OR 3.2, CI 1.33–7.69 for the middle age group and OR 14.1, CI 6.05–32.65 for the oldest age group). Diabetes was also positively associated with overweight and obesity and higher level of education completed, but these associations did not quite achieve significance (Table 2).

54.0% (CI 41.6–65.9) of the diabetic population had previously tested for diabetes. Progress through the cascade of care showed that 75.2% (CI 58.0–86.9) of the diabetic population who tested had also received a diabetes diagnosis, 92.6% (CI 80.3–97.5) of those diagnosed were treated, and 29.2% (CI 14.7–49.7) of those treated achieved adequate fasting blood glucose control (Fig. 2b). The largest relative gap was between receiving treatment and achieving control. The total gap in care, defined as the proportion of the total diabetic population that had not achieved adequate blood glucose control, was estimated at 88.1%.

Due to the low numbers of participants defined as having diabetes, independent variables association with progressing through the various stages in the diabetes care cascade, or achieving good control, were not examined.

Among people defined as being at overtly increased risk (due to increased age, high BMI, or smoking) prevalence of DM was 7.2%, compared to 2.0% in the rest of the population (Pearson chi2 32.2, *p* < 0.001). Significantly more people at high risk had been tested for diabetes than among those with lower risk (22.8% versus 11.3%, Pearson chi2 = 47.3, *P* < 0.001), and most had been tested for both DM and hypertension (21.0%) (Table 5).

## Discussion

This study reports the first nationally representative estimates of the burden of hypertension and diabetes, as well as care gaps among people in semi-autonomous state of Zanzibar. Overall, the prevalence of hypertension in our study was high, even in the youngest age group (25–34 years), while overall diabetes prevalence was relatively low.

For a poor state with a young population this is a serious concern as it poses substantial risk for high rates of CVD including stroke to be seen in the future. This is especially worrying as routinely collected data reveals that uncontrolled hypertension and diabetes already are leading causes of hospital admission for adults (11% in the group 13+ years), and diabetes and hypertension together with cerebrovascular accidents are leading causes of death for adults (23% of all causes) at hospitals [16].

No previous or subsequent cross-sectional studies have been conducted in Zanzibar to allow longitudinal analysis of trends, yet there are similar surveys conducted at several sites in the East African region. The Rwanda, Uganda, Kenya, Tanzania, and Malawian national STEPS surveys conducted at a similar time Zanzibar study, revealed lower prevalence of hypertension among their adult populations, and prevalence of diabetes was only higher in Malawi and Tanzania [17–21].

Our prevalence findings from Zanzibar are lower than the estimates from workplace screening of civil servants in Zanzibar (54% had hypertension, and 19% raised fasting blood glucose).^2^ This screening did not take into account the age of the participants or the participation rate. The proportion of participants with obesity was very high (33%), and there might have been a selection bias where younger, healthier individuals did not participate. Thus, that our study suggest lower national-level prevalence is not surprising.

In contrast to studies from elsewhere in sSA, in the present analyses there was no association found between employment, urban residency, or sedentary time, and having diabetes/hypertension. There was however a positive and independent association between older age, some formal schooling, obesity, raised fasting blood glucose levels, and having hypertension. For having diabetes, age and obesity were associated factors.

The lack of association between employment, residency, and being sedentary, and having hypertension or diabetes requires further exploration. The lack of association with sedentary time may be explained by those who are sedentary for > 3 h per day still being reasonably active for the remainder of the time (see Additional file 2). It may also be because our cut-point for sedentariness, which reflected other studies that found associations between sedentary time and cardiovascular diseases and risk factors [22, 23], is not appropriate for this population. We did not assess associations with wealth quintiles in this study as data were not available. However, it may be that wealth is a major determinant of diseases status in this population as has been found elsewhere [24] and be that formal employment as well as urban residency corresponds poorly with wealth.

In the longer term, effective strategies, that go wider than the delivery of treatments in health services, need to be put in place to prevent occurrence of cardiovascular disease risk factors [25]. In the short term, to cope with the high prevalence, especially of hypertension, health services urgently require investment. As the prevalence of hypertension in the subpopulation group considered to be at increased risk was twice that in the rest of the population, and the prevalence of diabetes almost four times that in the rest of the population, a first step in improving care for hypertension and diabetes could be to further improve testing and referral on to care in groups with risk factors that can easily be picked out by health care workers, i.e. people who are above 50 years, overweight/obese, or currently smoking. It is encouraging that a substantial proportion of people with these characteristics had been tested for disease, especially for hypertension; nevertheless, improvements could still be made in targeting individuals at high risk for screening.

The cascade analysis shows hypertension and diabetes care in Zanzibar to be sub-optimal especially compared to what can be achieved in well-functioning health care systems both in LMIC [3] and HICs [26]. Results were, however, better than those found in studies done in nearby Tanzania [20, 27]. It may be that the proximity to health facilities in Zanzibar facilitates access (98% of the population live within 5 km of a primary health care facility). Although prevalence of conditions was different, progress through the various stages between the hypertension and diabetes care cascade were quite similar. There are drop offs in care at all stages, reasons for this are likely to be complex, but may include interactions between individual, societal, cultural, financial, and health system factors including cultural beliefs, trust in health services, health literacy, health seeking behavior, quality of care, and health care workers performance.

That women were more often tested and detected in our study could reflect a more frequent use of the health care system among women, including for pregnancy and childbirth.

But still, 29.6% of women age 25–49 in the study had never had their blood pressure measured, despite a total fertility rate of 5.1 [28], and 99% of pregnant women attend antenatal care (ANC) at least once [16]. This may be explained by poor recall, however, despite BP measurement being an integral part of ANC, one out of five women attending ANC never get their BP measured, 3 and approximately 22% of women delivering at the tertiary level hospital did not have a single BP measurement done during admission [29].

Other factors associated with achieving cascade success for hypertension were being overweight and being in formal employment, perhaps due to those who are overweight being more aware of their health risk and those in employment being more able to access health care.

Among those on treatment, younger age alone was associated with achieving control; this is perhaps surprising as being older was associated with better progress through the rest of the cascade. Factors, including lower BP to start with in this younger age group, adequate treatment regimens, and better medication adherence need to be investigated to explain this difference.

Strategies to improve transit through the care cascade are likely to require complex system intervention studies. Single strategy interventions are unlikely to provide a solution; for example, mass screening for hypertension and/or diabetes at household level, with referral of suspect cases for further investigation and management at health facility level have in Kenya shown that as few as 23% of individuals screened positive for raised blood glucose took up referrals for care, even when consultation was free of charge [30], while 61% of individuals screened positive for raised blood pressure took up referral for care in Malawi [31].

Studies in high income countries have shown a relationship between mental ill health and increased prevalence of cardiovascular disease risk factors and/or poorer access to treatment [9, 32]. However, in this study, there was no association between symptoms of mental illness, hypertension or diabetes, and progress through the cascade of care. Performing sensitivity analysis using a less conservative threshold for diagnosis symptoms of mental ill health with a cut-off at 2/3 rather than 3/4, did not substantially alter the findings (Additional file 3). Given that these findings contrast with those seen in other situations, this result requires further exploration, including investigation of the GHQ-12 as a screening tool in this setting.

Following this STEPS survey and a Service Provision Assessment of health facility readiness, the Zanzibar Ministry of Health designed a NCD program, and established a NCD unit to implement the program. Activities included decentralizing selected NCD services to the higher primary care level, development of and training in guidelines to detect and treat hypertension and diabetes, and allowing facilities the freedom to order antihypertensive and anti-diabetic medications (change from a push- to a pull system), and community awareness activities. Program monitoring shows that these processes are being implemented, however challenges in providing the policy-ensured free services remain, including medicines and equipment (for example glucose strips) regularly being out of stock, 4 and data on treatment and patient outcomes remain sparse. Despite this there has been a positive development with increased overall funding for drugs, reducing the frequency of stock-out. Following the establishment of the NCD program, the national HIV/TB program in Zanzibar has introduced guidelines for screening for HTN and cardio-metabolic disease in acknowledgement of the common co-morbidity. The case management of HIV/TB patients with uncomplicated hypertension and diabetes is taking place at the HIV care clinics, while so far the clinics do not provide services for the general (TB or HIV negative) population. In-depth evaluation of these strategies with patient-focused outcomes is now needed.

### Limitations

The sample size for this study was small, however, it was adequately powered, the response rate was high, and there were few missing variables. The care cascade approach allows identification of gaps in health care services, but it does not provide understanding of why these gaps occur. Further research is needed to identify and address the reasons for lack of transit through the cascade. The data came from a cross-sectional study and although this provides a useful snapshot of health system performance, longitudinal studies are needed to provide a more nuanced assessment of changes to health policy.

Second, raised BP and FBG on testing on just one occasion are used synonymic with hypertension and diabetes, and control of hypertension or diabetes were defined using same methods and cut-off levels as for diagnosis. This is normal in surveys of this nature [3, 4], however it might lead to errors in prevalence estimates – in a clinical setting, raised BP would need to be confirmed on at least two consecutive visits, and raised fasting blood glucose may require an OGTT before a diagnosis of diabetes is made. According to the WHO/IDF consultation report on diagnosis of diabetes [33] only 75% of survey-detected raised blood glucose are confirmed to have clinical diabetes on repeat testing.

Finally, the amount of missingness in responses to household income questions meant income was not included as a covariate, and previous employment was not available. Although we have data on current employment and education status, which are indicators of socio-economic status, more granular data on wealth may lead to important issues of inequality and inequity in health and health care for the poorest being captured and paid due attention.

## Conclusion

Hypertension and diabetes represent largely silent epidemics that affects up to one in three adults in Zanzibar; the health system has had limited success in providing care along the continuum and ensuring diagnosis and control of blood pressure as well as raised blood glucose.

Our results showed that gaps in hypertension and diabetes control are universally large. Health system interventions must be designed to bridge these gaps by putting in place pro-poor strategies for access to primary health care including ensuring essential medicines and technologies for hypertension and diabetes, and ensure that primary care is able to provide for a wider range of non-communicable conditions and risk factor. Unless this is done, it is unlikely that good population level hypertension and diabetes control can be achieved in Zanzibar.

For this to happen, the government must spend more - but they also have to spend smarter. While enough is known about treatment of hypertension, diabetes, and other cardiovascular risk factors, little is known about how to implement these treatments in the specific low income contexts, and at scale [34]. To gain that understanding, qualitative as well as operational research is needed to identify local drivers of disease as well as successful control strategies.

## Supplementary information

**Additional file 1: Figure a1** Losses through the care cascade, separately for hypertension and diabetes, where reaching next step is depending on having reached previous step.

**Additional file 2: Table a1.** Test for correlation between total METs per week, and total hours per day spent being sedentary (− 0.008). or being sedentary > 3 h per day (− 0.027). Number of observations 2144.

**Additional file 3: Table a2** UV regression analyses of mental health, using a different cutoff of 3/4 and 2/3, and association with having diabetes or having hypertension or progressing through the hypertension care cascade.

**Additional file 4: Table a3.** UV regression analyses of individual variables and having hypertension, or having diabetes.

**Additional file 5: Table a4**. UV analysis for individual level variables, and progressing through the hypertension care cascade. Reaching next stage in the cascade depends on having reached previous step.

**Additional file 6; Table a5.** UV analysis of individual variables and having BP control, among all people with hypertension.

## Data Availability

The data that support the findings of this study are available from Zanzibar Ministry of Health. Restrictions apply to the availability of these data, which were used under license for the current study, and so are not publicly available. Data are however available from the authors upon reasonable request and with permission of Zanzibar Ministry of Health.

## Abbreviations

ANC: Antenatal care
BG: Blood glucose
BMI: Body Mass Index
BP: Blood pressure
CI: Confidence interval
CVD: Cardiovascular diseases
DBP: Diastolic blood pressure
DM: Diabetes mellitus
GDP: Gross domestic product
GHQ-12: General Health Questionnaire-12
HICs: High income countries
HIV: Human immunodeficiency virus
HTN: Hypertension
IDF: International Diabetes Federation
IFG: Impaired fasting glucose
LMICs: Low- and middle-income countries
METs: Metabolic equivalents
MH: Mental health
NCD: Noncommunicable diseases
OGTT: Oral glucose tolerance test
OR: Odds ratio
SBP: Systolic blood pressure
SEM: Standard error of mean
STEPS: Stepwise assessment of noncommunicable disease risk factors
TB: Tuberculosis
USD: US dollar
WHO: World Health Organization
ZAMREC: Zanzibar Medical Research Ethics Committee

## Footnotes

1 Tanzania 1996–-2016 shows 100% increase in the proportion of DALYs lost due to NCDs (all types). Data from the Global Burden of Disease project, IHME. https://vizhub.healthdata.org/gbd-compare/ accessed 10/25/2018.

2 Program data, National NCD program phase 1, accessed from NCD unit Zanzibar Ministry of Health February 2018 (number screened n=1996).

3 Data from the Safer Deliveries project, October 2017–- November 2018, of > 20,000 pregnant women.

4 Personal communications with Head of NCD unit, Zanzibar Ministry of Health, June 2018.
