# Supplementary material for: Hypertension and diabetes in Zanzibar – prevalence and access to care
Source: BMC Public Health. 2020 Sep 4;20:1352. doi: 10.1186/s12889-020-09432-8 (PMC7472575; doi:10.1186/s12889-020-09432-8)
Supplement: Supplementary file 1 — Additional file 1: Figure a1 Losses through the care cascade, separately for hypertension and diabetes, where reaching next step is depending on having reached previous step. [file 12889_2020_9432_MOESM1_ESM.docx]

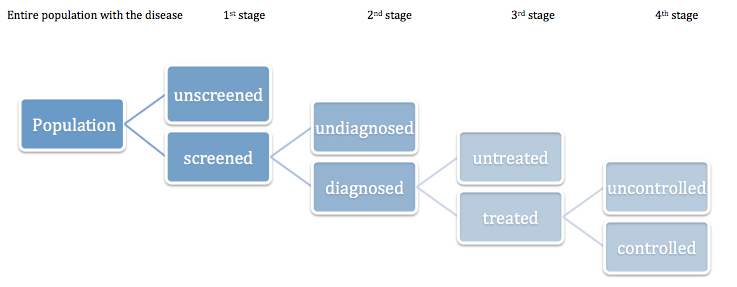


**Figure a1** Losses through the care cascade, separately for hypertension and diabetes, where reaching next step is depending on having reached previous step.
