# Supplementary material for: Hypertension and diabetes in Zanzibar – prevalence and access to care
Source: BMC Public Health. 2020 Sep 4;20:1352. doi: 10.1186/s12889-020-09432-8 (PMC7472575; doi:10.1186/s12889-020-09432-8)
Supplement: Supplementary file 2 — Additional file 2: Table a1. Test for correlation between total METs per week, and total hours per day spent being sedentary (− 0.008). or being sedentary > 3 h per day (− 0.027). Number of observations 2144. [file 12889_2020_9432_MOESM2_ESM.docx]

|  |  |  |  |  |
| --- | --- | --- | --- | --- |
|  |  | Total METs/week | Daily sedentary time | Sedentary > 3 hrs/day |
| Total METs /week |  | 1.000 |  |  |
| Daily sedentary time (hrs) |  | -0.008 | 1.000 |  |
| Sedentary time > 3 hrs/day |  | -0.027 | 0.758 | 1.000 |
|  |  |  |  |  |

**Table a1.** Test for correlation between total METs per week, and total hours per day spent being sedentary (-0.008)

or being sedentary > 3 hrs per day (-0.027). Number of observations 2144.
