# Supplementary material for: Hypertension and diabetes in Zanzibar – prevalence and access to care
Source: BMC Public Health. 2020 Sep 4;20:1352. doi: 10.1186/s12889-020-09432-8 (PMC7472575; doi:10.1186/s12889-020-09432-8)
Supplement: Supplementary file 3 — Additional file 3: Table a2 UV regression analyses of mental health, using a different cutoff of 3/4 and 2/3, and association with having diabetes or having hypertension or progressing through the hypertension care cascade. [file 12889_2020_9432_MOESM3_ESM.docx]

|  |  | |  | | |  | |  |
| --- | --- | --- | --- | --- | --- | --- | --- | --- |
|  | |  | |  |  | |  | |
| **HYPERTENSION** | |  | |  |  | |  | |
|  | |  | |  |  | |  | |
| **Having hypertension** | |  | | **OR** | **p-value** | | **95% CI** | |
| Mental health (cutoff 3/4) | |  | | 1.64 | 0.138 | | 0.85 - 3.14 | |
| Mental health (cutoff 2/3) | |  | | 1.02 | 0.93 | | 0.64 - 1.64 | |
|  | |  | |  |  | |  | |
| **Having been measured** | |  | |  |  | |  | |
| Mental health (cutoff 3/4) | |  | | 0.61 | 0.223 | | 0.28 - 1.35 | |
| Mental health (cutoff 2/3) | |  | | 0.85 | 0.643 | | 0.43 - 1.68 | |
|  | |  | |  |  | |  | |
| **Having been diagnosed** | |  | |  |  | |  | |
| Mental health (cutoff 3/4) | | | | 1.85 | 0.207 | | 0.71 - 4.82 | |
| Mental health (cutoff 2/3) | |  | | 1.61 | 0.213 | | 0.76 - 3.40 | |
|  | |  | |  |  | |  | |
| **Being on treatment** | |  | |  |  | |  | |
| Mental health (cutoff 3/4) | |  | | 0.51 | 0.153 | | 0.20 - 1.29 | |
| Mental health (cutoff 2/3) | |  | | 0.42 | 0.083 | | 0.16 - 1.12 | |
|  | |  | |  |  | |  | |
| **Having achieved control** | |  | |  |  | |  | |
| Mental health (cutoff 3/4) | |  | | 2.33 | 0.179 | | 0.68 - 8.06 | |
| Mental health (cutoff 2/3) | |  | | 1.29 | 0.626 | | 0.46 - 3.60 | |
|  | |  | |  |  | |  | |
| **DIABETES** | |  | |  |  | |  | |
|  | |  | |  |  | |  | |
| **Having Diabetes** | |  | | **OR** | **p-value** | | **95% CI** | |
| Mental health (cutoff 3/4) | |  | | 1.56 | 0.35 | | 0.62 - 3.94 | |
| Mental health (cutoff 2/3) | |  | | 1.53 | 0.231 | | 0.76 - 3.08 | |

**Table a2** UV regression analysis of mental health, using a different cutoff of 3/4 and 2/3, and association with having diabetes or having hypertension or progressing through the hypertension care cascade.

|  |  |  |  |
| --- | --- | --- | --- |
|  |  |  |  |
