# Supplementary material for: Hypertension and diabetes in Zanzibar – prevalence and access to care
Source: BMC Public Health. 2020 Sep 4;20:1352. doi: 10.1186/s12889-020-09432-8 (PMC7472575; doi:10.1186/s12889-020-09432-8)
Supplement: Supplementary file 4 — Additional file 4: Table a3. UV regression analyses of individual variables and having hypertension, or having diabetes. [file 12889_2020_9432_MOESM4_ESM.docx]

|  |  |  |  |
| --- | --- | --- | --- |
|  |  |  |  |
|  |  |  |  |
|  |  |  |  |
|  |  |  |  |

|  | **Having HTN** | | |  | **Having DM** | | |  |
| --- | --- | --- | --- | --- | --- | --- | --- | --- |
|  | OR | 95% CI | p-value |  | OR | 95% CI | p-value |  |
| **Sex** |  |  |  |  |  |  |  |  |
| Male | Ref |  |  |  | Ref |  |  |  |
| Female | 1.02 | 0.79-1.35 | 0.831 |  | 0.83 | 0.48-1.43 | 0.504 |  |
| **Age** |  |  |  |  |  |  |  |  |
| 20-34 years | Ref |  |  |  | Ref |  |  |  |
| 35-49 | 2.28 | 1.58-3.31 | <0.001 |  | 3.67 | 1.59-8.48 | 0.002 |  |
| 50-65 years | 9.37 | 6.21-14.13 | <0.001 |  | 12.02 | 5.23-27.60 | <0.001 |  |
| **Residence** |  |  |  |  |  |  |  |  |
| Rural | Ref |  |  |  | Ref |  |  |  |
| Urban | 0.85 | 0.65-1.12 | 0.254 |  | 1.66 | 1.00-2.74 | 0.049 |  |
| **Education** |  |  |  |  |  |  |  |  |
| No formal education | Ref |  |  |  | Ref |  |  |  |
| Some primary/secondary school | 0.44 | 0.33-0.60 | <0.001 |  | 1.04 | 0.57-1.90 | 0.903 |  |
| Secondary school or above complete | 0.50 | 0.36-0.70 | <0.001 |  | 1.39 | 0.72-2.68 | 0.325 |  |
| **Employment** |  |  |  |  |  |  |  |  |
| No formal or self employment | Ref |  |  |  | Ref |  |  |  |
| Self employed | 0.86 | 0.65-1.13 | 0.291 |  | 1.39 | 0.77-2.49 | 0.275 |  |
| Formally employed | 0.73 | 0.47-1.12 | 0.151 |  | 1.51 | 0.67-3.43 | 0.323 |  |
| **Tobacco use** |  |  |  |  |  |  |  |  |
| Never smoked | Ref |  |  |  | Ref |  |  |  |
| Former smoker | 1.83 | 1.11-3.00 | 0.017 |  | 1.24 | 0.53-2.91 | 0.620 |  |
| Current smoker | 0.86 | 0.51-1.47 | 0.590 |  | 1.00 | 0.43-2.31 | 0.996 |  |
| **BMI** |  |  |  |  |  |  |  |  |
| Normal or underweight | Ref |  |  |  | Ref |  |  |  |
| Overweight | 1.29 | 0.93-1.78 | 0.124 |  | 2.14 | 1.17-3.91 | 0.014 |  |
| Obese | 3.07 | 2.12-4.44 | <0.001 |  | 2.93 | 1.42-6.04 | 0.004 |  |
| **Raised Fasting Blood Glucose** | 3.1 | 1.93-4.98 | <0.001 |  | --- | --- | --- |  |
| **High colesterol** | 1.51 | 1.12-2.04 | 0.006 |  | 1.89 | 1.08-3.32 | 0.026 |  |
| **Sedentary for at least 3 hours/day** | 1.00 | 0.75-1.32 | 0.204 |  | 0.62 | 0.34-1.12 | 0.114 |  |
| **Mental illness present** | 1.41 | 0.83-2.39 | 0.204 |  | 1.26 | 0.51-3.14 | 0.620 |  |

**Table a3.** UV regression analysis of individual variables and having hypertension, or having diabetes.
