# Supplementary material for: Hypertension and diabetes in Zanzibar – prevalence and access to care
Source: BMC Public Health. 2020 Sep 4;20:1352. doi: 10.1186/s12889-020-09432-8 (PMC7472575; doi:10.1186/s12889-020-09432-8)
Supplement: Supplementary file 5 — Additional file 5: Table a4. UV analysis for individual level variables, and progressing through the hypertension care cascade. Reaching next stage in the cascade depends on having reached previous step. [file 12889_2020_9432_MOESM5_ESM.docx]

|  | **Screened** | | |  | **Diagnosed** | | |  | **Treated** | | |  | **Controlled** | | |
| --- | --- | --- | --- | --- | --- | --- | --- | --- | --- | --- | --- | --- | --- | --- | --- |
|  | OR | 95% CI | p-value |  | OR | 95% CI | p-value |  | OR | 95% CI | p-value |  | OR | 95% CI | p-value |
| **Sex** |  |  |  |  |  |  |  |  |  |  |  |  |  |  |  |
| Male | Ref |  |  |  | Ref |  |  |  | Ref |  |  |  | Ref |  |  |
| Female | 3.43 | 2.20-5.34 | <0.001 |  | 1.96 | 1.08-3.56 | 0.026 |  | 0.56 | 0.21-1.54 | 0.262 |  | 1.26 | 0.55-2.90 | 0.577 |
| **Age** |  |  |  |  |  |  |  |  |  |  |  |  |  |  |  |
| 20-34 years | Ref |  |  |  | Ref |  |  |  | Ref |  |  |  | Ref |  |  |
| 35-49 | 1.15 | 0.60-2.22 | 0.669 |  | 1.4 | 0.63-3.08 | 0.404 |  | 2.93 | 1.18-7.30 | 0.021 |  | 0.42 | 0.14-1.29 | 0.129 |
| 50-65 years | 1.39 | 0.73-2.66 | 0.321 |  | 2.78 | 1.27-6.09 | 0.011 |  | 4.36 | 1.69-11.20 | 0.002 |  | 0.12 | 0.04-0.38 | <0.001 |
| **Residence** |  |  |  |  |  |  |  |  |  |  |  |  |  |  |  |
| Rural | Ref |  |  |  | Ref |  |  |  | Ref |  |  |  | Ref |  |  |
| Urban | 2.29 | 1.44-3.62 | <0.001 |  | 0.85 | 0.51-1.41 | 0.525 |  | 1.12 | 0.55-2.29 |  |  | 0.54 | 0.26-1.16 | 0.113 |
| **Education** |  |  |  |  |  |  |  |  |  |  |  |  |  |  |  |
| No formal education | Ref |  |  |  | Ref |  |  |  | Ref |  |  |  | Ref |  |  |
| Some primary/secondary school | 1.46 | 0.92-2.32 | 0.110 |  | 0.73 | 0.40-1.33 | 0.306 |  | 1.02 | 0.41-2.52 | 0.963 |  | 1.00 | 0.42-2.40 | 0.995 |
| Secondary school or above | 3.00 | 1.73-5.20 | <0.001 |  | 0.68 | 0.34-1.34 | 0.266 |  | 1.12 | 0.42-8.01 | 0.817 |  | 1.22 | 0.47-3.17 | 0.681 |
| **Employment** |  |  |  |  |  |  |  |  |  |  |  |  |  |  |  |
| No formal or self employment | Ref |  |  |  | Ref |  |  |  | Ref |  |  |  | Ref |  |  |
| Self employed | 0.37 | 0.24-0.58 | <0.001 |  | 0.78 | 0.45-1.35 | 0.374 |  | 0.40 | 0.19-0.86 | 0.018 |  | 1.77 | 0.81-3.87 | 0.150 |
| Formally employed | 2.14 | 1.09-4.18 | 0.027 |  | 0.69 | 0.31-1.54 | 0.368 |  | 0.72 | 0.25-2.08 | 0.547 |  | 1.41 | 0.48-4.15 | 0.528 |
| **Tobacco use** |  |  |  |  |  |  |  |  |  |  |  |  |  |  |  |
| Never smoked | Ref |  |  |  | Ref |  |  |  | Ref |  |  |  | Ref |  |  |
| Former smoker | 0.50 | 0.23-1.07 | 0.075 |  | 1.55 | 0.69-3.47 | 0.290 |  | --- | --- | --- |  | --- | --- |  |
| Current smoker | 0.20 | 0.09-0.43 | <0.001 |  | 1.06 | 0.33-3.36 | 0.921 |  | --- | --- | --- |  | --- | --- |  |
| **BMI** |  |  |  |  |  |  |  |  |  |  |  |  |  |  |  |
| Normal or underweight | Ref |  |  |  | Ref |  |  |  | Ref |  |  |  | Ref |  |  |
| Overweight | 1.84 | 1.08-3.12 | 0.025 |  | 2.24 | 1.18-4.25 | 0.014 |  | 1.89 | 0.74-4.80 | 0.180 |  | 1.35 | 0.58-3.15 | 0.474 |
| Obese | 3.21 | 1.75-5.88 | <0.001 |  | 2.16 | 1.15-4.07 | 0.017 |  | 1.46 | 0.64-3.33 | 0.366 |  | 0.94 | 0.38-2.32 | 0.886 |
| **Previously diagnosed w diabetes** | --- | --- | --- |  | 5.25 | 1.66-16.60 | 0.005 |  | -- | --- | --- |  | --- | ---- |  |
| **Sedentary for at least 3 hrs/day** | 0.81 | 0.53-1.25 | 0.338 |  | 1.14 | 0.65-1.98 | 0.650 |  | 0.92 | 0.43-1.95 | 0.826 |  | 0.92 | 0.39-2.15 | 0.845 |
| **Mental illness present** | 0.75 | 0.31-1.84 | 0.533 |  | 2.24 | 0.91-5.53 | 0.081 |  | 0.52 | 0.20-1.33 | 0.169 |  | 2.31 | 0.67-7.97 | 0.184 |

**Table a4**. UV analysis for individual level variable, and progressing through the hypertension care cascade. Reaching next stage in the cascade depends on having reached previous step.
