# Supplementary material for: Hypertension and diabetes in Zanzibar – prevalence and access to care
Source: BMC Public Health. 2020 Sep 4;20:1352. doi: 10.1186/s12889-020-09432-8 (PMC7472575; doi:10.1186/s12889-020-09432-8)
Supplement: Supplementary file 6 — Additional file 6; Table a5. UV analysis of individual variables and having BP control, among all people with hypertension. [file 12889_2020_9432_MOESM6_ESM.docx]

|  | **Control among all HTN** | | | |
| --- | --- | --- | --- | --- |
|  | OR | 95% CI |  | p-value |
| **Sex** |  |  |  |  |
| Male | Ref |  |  |  |
| Female | 3.29 | 1.74-6.20 |  | <0.001 |
| **Age** |  |  |  |  |
| 20-34 years | Ref |  |  |  |
| 35-49 | 0.74 | 0.38-1.46 |  | 0.386 |
| 50-65 years | 0.49 | 0.23-1.05 |  | 0.066 |
| **Residence** |  |  |  |  |
| Rural | Ref |  |  |  |
| Urban | 0.85 | 0.49-1.47 |  | 0.558 |
| **Education** |  |  |  |  |
| No formal education | Ref |  |  |  |
| Some primary/secondary school | 0.94 | 0.49-1.08 |  | 0.847 |
| Secondary school or above complete | 1.46 | 0.71-3.00 |  | 0.306 |
| **Employment** |  |  |  |  |
| No formal or self employment | Ref |  |  |  |
| Self employed | 0.93 | 0.53-1.64 |  | 0.802 |
| Formally employed | 1.59 | 0.73-3.46 |  | 0.245 |
| **Tobacco use** |  |  |  |  |
| Never smoked | Ref |  |  |  |
| Former smoker | 0.31 | 0.12-0.84 |  | 0.021 |
| Current smoker | 0.17 | 0.04-0.76 |  | 0.020 |
| **BMI** |  |  |  |  |
| Normal or underweight | Ref |  |  |  |
| Overweight | 2.24 | 1.22-4.08 |  | 0.009 |
| Obese | 1.90 | 0.96-3.74 |  | 0.063 |
| **Previously diagnosed with diabetes** | 0.56 | 0.22-1.39 |  | 0.209 |
| **Sedentary for at least 3 hours/day** | 0.85 | 0.47-1.54 |  | 0.594 |
| **Mental illness present** | 1.79 | 0.76-4.20 |  | 0.181 |
| **Raised cholesterol** | 0.56 | 0.31-0.997 |  | 0.049 |
|  |  |  |  |  |

**Table a5.** UV analysis of individual variables and having BP control, among all people with hypertension.
